# Supplementary material for: Obtaining Nanostructured ZnO onto Si Coatings for Optoelectronic Applications via Eco-Friendly Chemical Preparation Routes
Source: Nanomaterials (Basel). 2021 Sep 24;11(10):2490. doi: 10.3390/nano11102490 (PMC8539447; doi:10.3390/nano11102490)
Supplement: Supplementary file 1 [file nanomaterials-11-02490-s001.zip › nanomaterials-1356977-supplementary.pdf]

Obtaining Nanostructured ZnO onto Si Coatings for Optoelectronic Applications via Eco-Friendly Chemical Preparation Routes

**Mirela Petruta Suchea [1,2,*], Evangelia Petromichelaki [3,4], Cosmin Romanitan [2], Maria Androulidaki [3],
Alexandra Manousaki [3], Zacharias Viskadourakis [3], Rabia Ikram [4,5], Petronela Pascariu [6,*] and George Kenanakis [3,*]**

[1] Center of Materials Technology and Photonics, Hellenic Mediterranean University, 71410 Heraklion, Crete, Greece
[2] National Institute for Research and Development in Microtechnologies (IMT-Bucharest), 023573 Bucharest, Romania; cosmin.romanitan@imt.ro
[3] Institute of Electronic Structure and Laser, Foundation for Research & Technology-Hellas, N. Plastira 100, 70013 Heraklion, Crete, Greece; epetromichelaki@physics.uoc.gr (E.G.P.); pyrhnas@physics.uoc.gr (M.A.); manousa@iesl.forth.gr (A.M.); zach@iesl.forth.gr (Z.V.)
[4] Physics Department, University of Crete, 71003 Heraklion, Greece; raab@um.edu.my
[5] Department of Chemical Engineering, University of Malaya, 50603, Kuala Lumpur, Malaysia; raab@um.edu.my (R.I.)
[6] "Petru Poni" Institute of Macromolecular Chemistry, 700487 Iasi, Romania
* Correspondence: mirasuchea@hmu.gr or mira.suchea@imt.ro (M.S.); dorneanu.petronela@icmpp.ro (P.P.); gkenanak@iesl.forth.gr (G.K.)

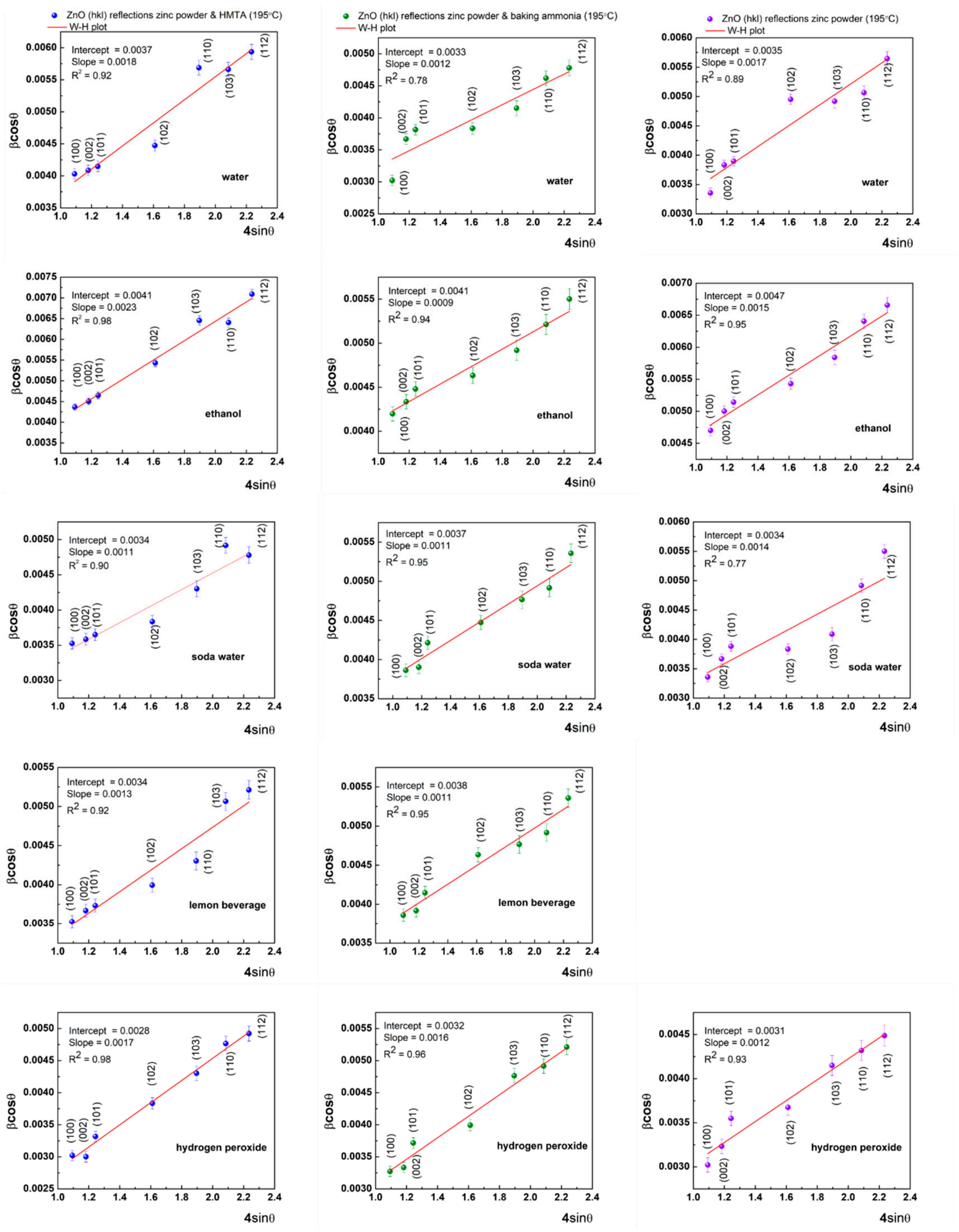

Figure S1. Wiliamson-Hall plot (red line) for the synthesized samples from Zn powder & Hexamethylenetetramine (HMTA) – column 1, Zn powder & baking ammonia – column 2 and Zn powder at 195°C in water, ethanol, soda water, lemon beverage and hydrogen peroxide, respectively - column 3. The value of the intercept and slope, as well as the fitting parameter R^2, that shows the goodness of fit are listed in each case.
